# Health systems challenges in cervical cancer prevention program in Malawi

**DOI:** 10.3402/gha.v8.26282

**Published:** 2015-01-22

**Authors:** Fresier C. Maseko, Maureen L. Chirwa, Adamson S. Muula

**Affiliations:** 1 School of Public Health and Family Medicine, College of Medicine, University of Malawi, Zomba, Malawi; 2 Prime Health Consulting and Services, Lilongwe, Malawi

**Keywords:** cervical cancer prevention, health system gaps, Malawi

## Abstract

**Background:**

Cervical cancer remains the leading cause of cancer death among women in sub-Saharan Africa. In Malawi, very few women have undergone screening and the incidence of cervical cancer is on the increase as is the case in most developing countries. We aimed at exploring and documenting health system gaps responsible for the poor performance of the cervical cancer prevention program in Malawi.

**Design:**

The study was carried out in 14 randomly selected districts of the 29 districts of Malawi. All cervical cancer service providers in these districts were invited to participate. Two semi-structured questionnaires were used, one for the district cervical cancer coordinators and the other for the service providers. The themes of both questionnaires were based on World Health Organization (WHO) health system frameworks. A checklist was also developed to audit medical supplies and equipment in the cervical cancer screening facilities. The two questionnaires together with the medical supplies and equipment checklist were piloted in Chikwawa district before being used as data collection tools in the study. Quantitative data were analyzed using STATA and qualitative in NVIVO.

**Results:**

Forty-one service providers from 21 health facilities and 9 district coordinators participated in the study. Our findings show numerous health system challenges mainly in areas of health workforce and essential medical products and technologies. Seven out of the 21 health facilities provided both screening and treatment. Results showed challenges in the management of the cervical cancer program at district level; inadequate service providers who are poorly supervised; lack of basic equipment and stock-outs of basic medical supplies in some health facilities; and inadequate funding of the program. In most of the health facilities, services providers were not aware of the policy which govern their work and that they did not have standards and guidelines for cervical cancer screening and treatment.

**Conclusion:**

Numerous health system challenges are prevailing in the cervical cancer prevention program in Malawi. These challenges need to be addressed if the health system is to improve on the coverage of cervical cancer screening and treatment.

Cervical cancer is the most common cancer among women in sub-Saharan Africa (1, 2). It is the leading cause of cancer deaths among women in sub-Saharan Africa (3, 4). However, cervical cancer is preventable and curable, at low cost and low risk, when screening to facilitate the timely detection of early precursor lesions in asymptomatic women is available together with appropriate diagnosis, treatment, and follow-up (5). According to the 2014 Africa Cervical Cancer Multi Indicator Incidence and Mortality Scorecard, out of the 20 countries globally with the highest incidence of cervical cancer, 16 are African countries (6). In sub-Sahara, the estimated overall standardized incidence rate is 31 per 100,000 women (1, 7). It is estimated that 70,722 cases occur annually in the sub-Saharan region, which accounts for a quarter of all female cancers (1). Although eastern Africa region has the highest incidence of cervical cancer, Malawi and Mozambique are globally known to have the highest age-standardized incidence rate of 75.9 and 65.0, respectively (6).

Early cervical cancer screening can detect cervical intraepithelial grades 2 or 3 which are precancerous lesions and, if treated, can prevent women from developing invasive cervical cancer. This calls for the health systems to implement well-organized secondary prevention programs that should provide screening for all women as well as treatment of detected abnormalities (8, 9). The health systems need to implement programs that provide affordable and acceptable cervical cancer screening and treatment services which are accessible to ensure coverage and timeliness to the target population. These services should be provided by skilled and competent health workers who are knowledgeable and skilled. The programs should also provide services which are sustainable and safe to providers, patients, and the environment (9). Research and experience show that well-organized screening and treatment programs are effective in cervical cancer prevention (10). Paradoxically, most health systems in sub-Saharan Africa do not have screening programs for early detection of precancerous lesions despite having increased numbers of cervical cancer cases diagnosed annually in their health facilities. Those handful health systems that have screening and treatment programs are known to be faced with numerous challenges to the extent that their cervical cancer screening coverage is far below the recommended 70% of the targeted population within the first 10 years of implementation (4). Most of cervical cancer screening and treatment programs in sub-Saharan Africa have failed because of lack of goals or existing protocols. Bradley and her colleagues argue that inadequate staff training in screening, treatment, referral, and record keeping are some of health system challenges which have contributed to failure of health systems in implementing effective cervical cancer screening and treatment programs. Some health systems in the region do not have basic infrastructure to carry out cervical screening and treatment and as such cannot offer cervical cancer screening and treatment (11).

In Malawi, where on average 3,684 women are diagnosed with cervical cancer annually (12), it is estimated that 85% of those women who develop cervical cancer die from the malignancy (7, 13). However, cervical cancer screening and treatment program has been in existence for the past two and a half decades in the country (12). The program was started in few districts with financial support from different international non-governmental organizations, but currently is under full control of the Ministry of Health. The program has been rolled out to all central and district hospitals. Some districts have also managed to roll out the program to their health centers. In all districts, cervical cancer screening and treatment activities were integrated into reproductive health services. Despite the effort, very few women have undergone screening and the incidence of cervical cancer in Malawi is on the increase as is the case in most developing countries (14). Much of the focus of studies done in Malawi has been to explore individual and community factors that impede women from cervical cancer screening (15–17) yet there are numerous health system challenges in health care delivery in Malawi. Shortage of adequately trained and available health professionals, inadequate infrastructure (including basic equipment, transport, and communication), and insufficient stocks of drugs and medical supplies have been documented as some of the perpetual constraints to the health system in Malawi (18, 19). In view of this, we carried out a study aiming at exploring and documenting health system gaps responsible for the poor performance of the cervical cancer screening and treatment in Malawi. Specifically, we wanted to assess the knowledge, attitude, and practice of the health workers who are directly involved in the provision of cervical cancer screening and treatment, and to identify health system challenges that exist in the provision of cervical cancer screening and treatment in Malawi.

## Methods

### Study design

The study was an exploratory cross-sectional, mixed methods designed, using both quantitative and qualitative approaches.

### Sampling

The sample size for the study was determined using Raosoft sample size calculator (http://www.raosoft.com/samplesize.html) (20). Given that there are 29 health districts in Malawi, expecting response distribution of 99% and allowing 5% margin of error at 95% confidence interval, the required sample size was calculated to be 11. The sample size was then increased to 14 in anticipation of non-response (21). To ensure the random selection of the districts, each of the 29 administrative health districts was assigned a number from 1 to 29 against its name, arranged in alphabetical order. Using Graphpad Software, 14 randomly selected numbers were generated, each within the value range of 1 and 29 which were then matched to select the 14 districts. The following districts were selected: Chiradzulu, Dedza, Karonga, Lilongwe, Mchinji, Mulanje, Mwanza, Mzimba North, Mzimba South, Nkhata Bay, Nsanje, Phalombe, Rumphi, and Zomba. All health professionals providing cervical cancer prevention services in these 14 randomly selected administrative health districts constituted the sample for the health professionals.

### Data collection

Two semi-structured questionnaires were developed. One questionnaire targeted cervical cancer service providers and the other was for district cervical cancer program coordinators. The themes of both questionnaires were based on World Health Organization (WHO) health system frameworks (22). The framework has six pillars as building blocks of a health system which are health services delivery; health workforce; health information systems (HIS); leadership and governance; essential medical products; vaccines and technologies; and financing. The questionnaire for the service providers also included questions on health workers’ knowledge, attitude, and practice. Other questions were on providers’ workload, supervision, and data collection and management. It also captured information on medical products and stock-outs.

The questionnaire for the district cervical cancer coordinators included questions on how services are delivered, who provide the service and their capacity to provide the services, how supervision is done, how data on cervical cancer flow from the facilities to the district then to the National Cancer Registry, and how referral is managed within the district. The questionnaire also collected information on how the pharmaceutical products are managed. The questionnaire also contained questions on how the cervical cancer prevention programs were financed and governed in the district. A checklist was also developed to audit the medical supplies and equipment that the cervical cancer screening and treatment clinics had in stock.

Cervical cancer service providers and district cervical cancer coordinators filled the questionnaires under the guidance of trained research assistants. The service providers also provided information on the medical supplies and equipment their clinic had. The two questionnaires together with the medical supplies and equipment checklist were piloted in Chikwawa district before being used as data collection tools in the study.

### Data management and analysis

Completed questionnaires were checked for completeness and consistency by data collection supervisors. Using Epi Info™ 7 (7.1.2) for windows, a database was created and data entered which were later imported into STATA version 11 for window for data cleaning and analysis. Descriptive statistics were computed to summarize participant characteristics. Qualitative data were loaded in NVIVO 9 and thematic content analysis, which involved a process of coding and identifying emerging themes (23), was carried out.

### Ethical consideration

Ethical approval to conduct the study was obtained from the College of Medicine Research and Ethics Committee (COMREC) of the University of Malawi in November 2012. Permission was also obtained from the Ministry of Health Zone Support Offices and District Health offices to conduct the study in their health facilities. To maintain the privacy of the participants, each interview was done in an examination room in the absence of the health service provider. To maintain confidentiality, information obtained from participants was handled only by those authorized.

## Results

All 41 service providers from 21 health facilities and 9 district health coordinators, that were approached, consented and participated in the study. Of the 21 facilities, one was a central hospital, 7 health centers, and 13 district hospitals. Of the 41 service providers, 29 came from district hospital while only 5% were interviewed from the central hospital. The majority (38 participants) of the providers were female nurse technicians, aged between 25 and 63, and were married.

### Service delivery

Only 7 of the 21 health facilities we conducted interviews provided both screening and treatment of cervical cancer. Patients were reported to be referred from those facilities with no treatment equipment to the districts or central hospitals when they have been diagnosed with cervical cancer. Only one health facility was open throughout the week and six opened from Mondays to Fridays for cervical cancer screening. Six health facilities opened once a week for cervical cancer screening while eight opened twice.

On average, 10 women were screened per clinic day. This was on the lower side as commented by 88% of the service providers. According to them, the possible reasons for the lower numbers were lack of awareness about the disease and the services. One participant said

… women are not aware that we started VIA (visual inspection with acetic acid) here despite the clinic being in operational for about five months. And even the District Health Management Team (DHMT) has never taken the initiative to allow us to go on a sensitization campaign to make sure that we reach out to the women in the communities. Every time we remind management we are told that what we are asking was not put in the district implementation plan and as such there isn't any money allocated to that exercise ….

Other reasons mentioned by district coordinators included lack of personnel who are committed to their work, lack of resources such as acetic acid (vinegar), the fact that the clinic opens only one day in a week, women afraid of knowing their cervical cancer status, and unwillingness of the women to come for the screening despite knowing that the services are available.

… yea indeed some are aware of the services we provide but they just don't want to come for cervical cancer screening. There are times when women come for screening but we send them back because all trained providers are busy with work in their wards … sometimes we send them back because we do not have the resources. You know there are times when we run short of supply of vinegar or even cotton wool so in such times we cannot do the screening ….

### Human resources

Out of the 14 districts we visited only 11 had cervical cancer district coordinators and 9 consented to participate in the study. Six coordinators reported of not having staff readily available to provide the services in their districts and two of them pointed out that the distribution of providers is not balanced between the urban and the rural facilities. The distribution favored urban centers. All service providers were trained health professionals; however, 35 out of the 41 had undergone initial training on cervical cancer screening and treatment provision. Six district coordinators reported that their providers were engaged in continuous professional development (CPD) in cervical cancer prevention. Study participants in two districts reported to have CPD monthly while the other four, quarterly. It was noted that no facility had a service provider who was allocated specifically for cervical cancer screening and treatment. In all health facilities, there were no service provider who was specifically and permanently allocated to work in the cervical cancer screening and treatment clinic. Service providers were assigned from other departments to provide the service at the cervical cancer clinic.

### Clinical supervision

Both service providers and district coordinators were asked about supervision in terms of the frequency and how it is done in their facilities. It was reported that supervision is done in some of the facilities. Four of the nine district coordinators reported to carry out supervisions in their districts, which was verified by 19 service providers. When asked how supervision is conducted in the district, one of the district coordinators said that it is their District Nursing Officer (DNO) who organizes the supervisions for them.

… she is the one who arranges transport for us and when we get to the clinic, we check if all the instruments needed for the procedures are available and we observe the actual screening process. Where a provider has made a mistake we give her the technical support needed to rectify the error. Sometimes we do the screening ourselves so that the provider sees how a procedure is supposed to be done … in most cases I go to the supervision with the matron or if she is not around, I go with one of the three trained providers I work with here at the district ….

The majority of the providers (24) appreciated the importance of supervision and wished they were frequently and timely supervised. Almost district coordinators in all districts reported that supervision was not done as scheduled. When asked why it is not done as scheduled or not done at all, the district coordinators pointed out the lack of transport due to unavailability of vehicles or fuel as the major contribution to poor or no supervision.

### Essential medical products, vaccines, and technologies

On drugs and supplies, only 48% of the service providers reported to have no stock-out of pharmaceutical products (glacial acetic acid, cotton, gloves, gauze pieces, bleaching powder, detergent, and distilled water) throughout the year in their facilities. Two of the nine coordinators reported to have no stock-outs of pharmaceutical products throughout the year in their districts. Fifty-two percent reported to have one or more stock-outs which could last for a week or more. An audit of equipment, consumables, and the stationery required to be present in VIA testing facility showed that most facilities had the basic necessary equipment and consumables to carry out VIA testing (Table 1). However, most of the facilities did not have the required equipment and consumables to conduct more advanced procedures such as cervical biopsy and cryotherapy. For instance, only seven facilities had working cryotherapy machines while 8 of the 21 health facilities had NO_2_ cylinders of which only five health facilities had cylinders filled with NO_2_ at the time of the survey.

**Table 1:** Number of health facilities with equipment, consumables, and stationery needed for VIA

Equipment/instrument	*n*=21	Consumable	*n*=21	Stationery	*n*=21
Screening centers					
Examination bed	21	Glacial acetic acid	18	Flipchart	0
Spotlight	21	Cotton	21	Posters	11
Instrument trolley	21	Gloves	21	VIA−ve cards	9
Autoclave	12	Gauze pieces	18	VIA +ve cards	9
Spare bulb	9	Bleaching powder	14	VIA register	21
Cusco's specula	21	Detergent	17	VIA +ve register	19
Sponge holding forceps	21	Distilled water	12	Reporting forms	21
Bowls/galipot	20			Checklist	10
Trays	20				
Instrument drum	20				
Buckets	21				
Measuring cylinder	7				
Container for acetic acid	21				
Additional requirement for Colposcopy					
Colposcope	4	Monsel's paste	2	Colposcopy register	14
Punch biopsy forceps	5	Vaginal packs	8	Reporting forms	21
Endocervical forceps	21	Potassium iodide	0		
		Iodine crystals	8		
		Formalin Solution	0		
		Container for biopsy	5		
Additional requirement for cryotherapy					
Cryotherapy machine	7	Filled up cylinder	5	Consent form	6
Cryoprobes	8	KY jelly	1		
NO_2_ cylinder	8				
Cylinder holder	7				

### Health management information system

All district coordinators except one did not know the number of women who are at risk of developing cervical cancer, those that need the services and those that have utilized the services in their districts. According to the district cervical cancer coordinators, all facilities are supposed to submit quarterly reports to the Zone Health Support Office (ZHSO). Asked how many cervical cancer reports they had submitted in the previous year, four of the districts coordinators reported to have submitted in all the quarters, one had only submitted twice and two districts once. The other four districts had never submitted any report. The coordinators were also asked if at all they have clear standards and guidelines for data collection and reporting procedures. They were also asked if the providers are trained on data collection, analysis, and reporting. All coordinators reported to have the data collection and reporting standards and guidelines. However, only two coordinators reported that providers in their districts underwent training regarding data collection, analysis, and reporting. Coordinators also reported that staff in their districts perceived and appreciated the importance of HIS. Asked if the coordinators give feedback to the service providers who generate the data, it was noted that over half of the coordinators do not.

Service providers were asked if at all there are any medical records that they complete after attending to patients or clients and if they were happy filling them. All services providers admitted filling medical record and that they are happy filling them as it is part of their job. However, five service providers reported to have not been trained on how to complete the medical records. On feedback, 25 of the 41 providers reported getting feedback from their supervisors on how they are doing on medical record filling. The district coordinators were also asked if the data they collect on cervical cancer screening and treatment are ever incorporated in the basic planning and management of their activities. Only three of the coordinators reported to incorporate the data they collect in their management and planning of cervical cancer activities in their districts.

### Finances

As reported by all district coordinators, there is no user fee to access cervical cancer screening and treatment in public health facilities in their districts. However, women are required to purchase a health passport, if they do not have one. At one district hospital, women had to contribute money to buy vinegar for the VIA as the facility had run out of stock. No district coordinator respondent as to how much his or her cervical cancer prevention program was allocated in that year's financial budget. Asked if whatever was spent on cervical cancer in the previous financial year was enough, more than half of the respondents said the funding was insufficient for effective implementation of the program.

### Leadership and governances

Malawi does not have a stand-alone policy for cervical cancer. Cervical cancer is covered under the reproductive cancer theme in the sexual and reproductive health rights (SRHR) policy. The service providers were asked if they were aware of the SRHR policy. Only 12 providers reported to be aware of the policy. Five service providers reported to be aware of the national service delivery guidelines for cervical cancer and 11 of the participants reported to have the national therapeutic guidelines with standardized treatments for cervical cancer in their clinics. Only in two districts cervical cancer coordinators reported to have updated clinical standards and guidelines while three had national therapeutic guides with standardized treatments available in all facilities in their districts.

### Improvements

Districts coordinators and service providers were asked what they thought could be done to improve delivery of cervical cancer screening and treatment. All were in agreement that there is need for intensive community sensitization about cervical cancer, scaling up the program to all health centers and training more providers who can do cervical cancer screening and treatment. They also pointed out the need to have cervical cancer clinics running from Monday to Friday as is the case with other reproductive health services. Providers also pointed out the need of allocating a room specific for cervical cancer screening and treatment as in most facilities the services were offered in family planning rooms which were always busy. Some providers also mentioned of providing the necessary material needed for the screening and treatment which most facilities in the districts were in short supply.

## Discussion

There are numerous health system challenges in the cervical cancer prevention in Malawi which are directly and indirectly contributing to the low coverage of cervical cancer screening and treatment. Our findings show numerous health system challenges mainly in areas of health workforce and essential medical products and technologies. Sufficiency and distribution of human resource is important in determining access to health care (25). However, our findings are showing that the cervical cancer prevention program in Malawi has insufficient workforce, which is also not fairly distributed. This is consistent with the findings of research by Palmer on human resource for health in Malawi (26). Considering the fact that some of the service providers had not undergone formal training to offer cervical cancer prevention services, and also considering how this workforce is managed, it is questionable if this workforce works in ways that are responsive, fair, and efficient to achieve the best outcomes. Lack of adequate service providers in the facilities also affected directly and indirectly the quality of the services being offered. With very few service providers, facilities could not manage to operate the cervical cancer prevention clinics on daily basis. This minimized the numbers of women utilizing the services.

We observed some challenges in the management of the program at district level. Some of the districts did not have coordinators to manage the cervical cancer screening program. With no coordinator in the district, there is no one to plan and conduct supportive technical supervision in the health facilities. Such districts have also no one to do the reporting which is crucial for planning, monitoring, and evaluation of the program at both district and national level. Most of the service providers were not trained on how to fill in medical records. With such type of service providers in the cervical cancer screening facilities, it is unlikely that they collect any data at all and for those who collect data, the data collected is of poor quality. With no monthly or quarterly reports, as was the case with some districts in the study, it is not possible for districts or the ZHSOs to know the needs of the facilities in terms of drugs and medical supplies, referral system, human resources, and infrastructure. This could also explain why cervical cancer data are not incorporated in the district HIS to inform the formulation of the district implementation plans and budgets.

Reports of unavailability of drugs and key medical consumables are very common in Malawian public health facilities (18, 27). Our findings indicated that less than half of the participants reported to have no stock-out of pharmaceutical products throughout the year in their facilities. This is expected in a health system which is characterized by a heavy burden of disease (28), where health services and medical supplies are free at a point of delivery (29, 30) and yet government health expenditure has always been less than 10% of the total annual budget (31, 32). With little funding, it is not possible for the district health offices to procure equipment and medical consumables to conduct more advanced procedures such as cervical biopsy and cryotherapy as these are expensive (33).

Health financing is a cross-cutting health system function affecting performance of all the other functions (29, 34). District coordinators could not explain how much was allocated for the cervical cancer prevention programs in their districts. This is not surprising as district funding in Malawi health system is not disease based. Although it was reported by both the service providers and the district coordinators that services are free, there are some indirect costs which service users incur to access them. These include transportation to the cervical cancer screening facilities and referral centers (35) and purchasing of health passport which is a requirement for every patient who is accessing the services. The health system in Malawi faces absolute and relative inadequacy of financing resources to adequately fund essential health package (EHP) services (24) and non-EHP services.

## Conclusion and recommendation

There are numerous health system challenges which are prevailing in the cervical cancer prevention program in Malawi. This study has highlighted some of these health system challenges which include lack of adequate, well-trained, evenly distributed, and well-supervised workforce. The study has also highlighted stock-outs of basic equipment and medical consumables required for screening and treatment of cervical cancer in the health facilities. In general, we recommend that the Malawi health system should train more cervical cancer prevention service providers who should be fairly distributed between urban and rural areas. It should ensure that the service providers are technically supported through periodical supervision. Furthermore, there is a need for the health system to strengthen the HIS so that it facilitates the flow of information from the cervical cancer screening and treatment facilities to the districts and from the districts to the facilities. The health system should intensify sensitization campaigns targeting especially women in the rural who have poor knowledge and little access to cervical cancer prevention services. The messages in the campaign should be organized in such a way that they take into account the educational, sociocultural, and religious barriers which are hindering the women to access cervical cancer prevention services.

The Ministry of Health should make deliberate efforts to ensure that cervical cancer screening and treatment services are rolled out to all public health facilities so that more women have access to the services. It also has the responsibility to make sure that all screening and treatment facilities have the basic infrastructure, equipment, and medical consumables required to offer quality and affordable cervical cancer screening and treatment services. In order to achieve this, there is need for the Government of Malawi to honor its commitment to the Abuja declaration. It needs to allocate at least 15% of its national annual budget to health. This would also improve the district health funding thereby increasing the amount of funding allocated for cervical cancer screening and treatment.
